# Current status and comparison of personal characteristics of freshmen undergraduates majoring in clinical pharmacy in southwest China

**DOI:** 10.3389/fmed.2025.1597812

**Published:** 2025-10-29

**Authors:** Yang Liu, Hang Zheng, Junhao Jiang, Bo Yan

**Affiliations:** ^1^School of Public Health, Chongqing Medical University, Chongqing, China; ^2^School of Pharmacy, Chongqing Medical University, Chongqing, China

**Keywords:** clinical pharmacy, personal characteristics, southwest China, undergraduate freshmen, course development

## Abstract

**Objective:**

We aimed to analyze and compare the personal characteristics of undergraduate freshmen enrolled in clinical pharmacy across colleges and universities in southwest China. The goal was to delineate the current composition of the freshmen cohort and offer insights for targeted teaching strategies.

**Methods:**

From January 2024 to February 2024, a questionnaire survey was conducted among first-year undergraduate students majoring in clinical pharmacy in eight universities in the Sichuan Province, Yunnan Province, Guizhou Province, and Chongqing City, China. The survey covered basic information and various scales.

**Results:**

Overall, 535 first-year undergraduate students, majoring in clinical pharmacy, completed the survey. Significant regional differences in personal characteristics existed (*p* < 0.05). While the respondents had similar personality traits, differences existed in their achievement goals, motivations, interests, and expectations (*p* < 0.05).

**Conclusion:**

This study provides suggestions for optimizing training programs to improve the education quality in clinical pharmacy, students’ practical abilities, and career expectations. Moreover, it offers valuable insights for refining training strategies and educational practices in clinical pharmacy, laying a solid foundation for the cultivation of future professional personnel.

## 1 Introduction

Clinical pharmacy, a patient-centered discipline, was developed in the United States in the mid-20th century. The American College of Clinical Pharmacy defines it as a pharmaceutical field focused on rational medication practice (1, 2). Its primary purpose is to empower pharmacists to provide direct pharmaceutical monitoring services to patients, enhancing drug treatment outcomes, preventing adverse reactions, and reducing medication errors (2).

In the United States, aspiring licensed clinical pharmacists undergo at least a 2-year preparatory course post high school and an entrance examination to enter a 4-year professional course at pharmacy school. Upon completion of the 4-year program, graduates are eligible for licensure (3–5). This is called a PharmD program. Following graduation, individuals can pursue employment in various settings, such as community pharmacies or pharmaceutical companies. Alternatively, they may opt for 1 or 2 years of resident pharmacist training to specialize as general or clinical pharmacists, respectively (6).

Some researchers identified key characteristics of professionalism in pharmacy students, including responsibility, commitment to excellence, respect for others, honesty and integrity, and caring and compassion. These traits should guide students’ actions and behaviors in their interactions with patients, colleagues, and society (7, 8). Pharmacists’ personality traits, confidence, need for approval, and risk aversion have been shown to influence their service provision (9, 10).

The development of clinical pharmacy in China traces back to 1978 when Wang Guofen and colleagues published a seminal report advocating for the initiation of clinical pharmacy pilot programs in the country. Subsequently, in the 1980s, China embarked on the exploration of clinical pharmacy, establishing key specialties within hospital settings (11).

Currently, 54 Chinese colleges and universities offer 5-year undergraduate majors in clinical pharmacy, with over 40 institutions providing master’s degree programs with a specialization in clinical pharmacy (12). Additionally, training graduate and doctoral candidates in clinical pharmacy has gained momentum.

In China, aspiring clinical pharmacists typically undergo 5 years of undergraduate education. Following graduation, they must attend a clinical pharmacist training base for 6 months to 1 year and pass the clinical pharmacist licensing examination to obtain a practicing certificate (13).

Originally focused on preparing pharmaceutical practitioners for industry or research (14), pharmaceutical education in China has shifted in response to the increasing demand for well-trained pharmacists equipped with clinical knowledge and skills to enhance medical care quality (15). Over nearly 40 years, clinical pharmacy has developed considerably in China. Current undergraduate programs in clinical pharmacy aim to cultivate professionals proficient in basic clinical pharmacy knowledge, theories, skills, and innovative thinking capable of providing pharmaceutical care with rational drug use at its core. While research is primarily centered on teaching methods, practical skills, and pharmaceutical care, a substantial knowledge gap exists regarding students’ personality traits and characteristics (16, 17).

According to previous research, in addition to knowledge and skills, the professional qualities of pharmacists (such as responsibility and honesty) and personal character are also crucial. Currently, clinical pharmacy education in China is developing rapidly, but there is still a gap in research on students’ personality traits compared to other countries like the United States. This study aims to fill this gap by investigating the training needs, personality characteristics, and career planning of new clinical pharmacy students in the southwest region of China.

Southwest China currently has eight universities providing clinical pharmacy education, including the Sichuan University and Dali University, along with six medical colleges. This accounts for 14.8% of all undergraduate clinical pharmacy programs in the country. Our study aimed to understand the training needs of clinical pharmacy students in the country. Our research focused on understanding the training needs of clinical pharmacy students in China, examining their personality characteristics, learning orientations, decision-making methods, and career plans.

## 2 Methods

### 2.1 Respondents

The survey targeted freshmen from eight universities offering undergraduate clinical pharmacy majors across four provinces and cities in southwest China: Sichuan Province, Yunnan Province, Guizhou Province, and Chongqing Municipality. Universities in the Tibet Autonomous Region were excluded as they do not offer undergraduate clinical pharmacy majors. The inclusion criteria are: (1) the surveyed university in southwest China, (2) it offers an undergraduate major in clinical pharmacy, (3) participants are first-year undergraduates, and (4) they agree to participate. Participating universities included Sichuan University, Southwest Medical University, North Sichuan Medical College, Kunming Medical University, Dali University, Guizhou Medical University, Zunyi Medical University, and Chongqing Medical University. Exclusion criteria: (1). Students who are not in their first year of undergraduate study in the clinical pharmacy major; (2). Individuals who cannot understand the questionnaire content or cannot complete the survey independently due to language, cognitive, or mental disorders; (3). Participants who have not completed most of the questionnaire items (such as questionnaire completion rate below 80%).

### 2.2 Development of the questionnaire

Based on a review of literature and policies related to the development of clinical pharmacy majors, a questionnaire was compiled to assess the personal characteristics of freshmen in southwest China. Before distributing the formal questionnaire, we conducted a small-scale preliminary survey. Through reliability and validity tests and feedback questions, we deleted some items with low reliability and validity, added incomplete items, modified items with unclear answers, and finally conducted reliability and validity tests again to finally form the formal questionnaire. The questionnaire includes sections on basic personal information, students’ motivation for pursuing the clinical pharmacy major, their interest in clinical pharmacy practice, and their expectations for future careers. Additionally, it incorporates the Big Five Personality traits using a simplified scale, the Achievement Goal Questionnaire to examine learning orientation, and the Rational-Experiential Inventory to investigate decision-making styles (18). In the field of personal development, the Big Five Personality trait is used to help individuals understand their personality traits and guide their personal growth and career development planning. The Achievement Goal Questionnaire mainly explores the relationship between achievement goals and individual expectations, attributions, motivation, and behavior, revealing the intrinsic mechanism by which motivation is converted into behavior. The Rational-Experiential Inventory is mainly used to quantify the preference differences between two cognitive modes, rational analysis and intuitive experience, in the process of information processing and decision-making for individuals. The scale’s reliability coefficient (Cronbach’s alpha) was 0.904. The scoring was based on a five-point Likert scale: 1 for strongly disagree, 2 for disagree, 3 for uncertain, 4 for agree, and 5 for strongly agree. Higher scores indicate greater motivation for learning clinical pharmacy, interest in clinical pharmacy practice, and career expectations among the interviewed freshmen.

### 2.3 Data collection

Overall, 693 online questionnaires were distributed to all admitted freshmen using Questionnaire Star software,^1^ resulting in 535 returned questionnaires. Among them, 535 were deemed valid, achieving an effective recovery rate of 77.2%.

### 2.4 Data analysis and statistics

Statistical analysis was performed using SPSS27.0 software.^2^ The basic socio-demographic characteristics of the survey respondents were described using frequency and composition ratio (%). Differences among the samples were investigated using single-factor analysis of variance, independent sample *t*-tests, and chi-square tests, with significance indicated by *p* < 0.05. Correlation analysis and binary logistic regression analysis were employed to explore sample correlations, with significance set at *p* < 0.05.

## 3 Results

### 3.1 Basic information of the respondents

We surveyed a total of 535 freshmen enrolled in clinical majors across colleges and universities in southwest China. Table 1 illustrates the distribution of respondents: 216 from universities in the Sichuan Province, 127 from universities in the Guizhou Province, 127 from universities in the Yunnan Province, and 65 from universities in Chongqing. Among the respondents, 291 were female, comprising 54.40% of the sample. The majority of respondents fell within the age group of 18 and 19 years old, constituting 89.00% of the total.

**Table 1:** Basic information of respondents.

Variable	Total (*n* = 535)	Yunnan (*n* = 127)	Sichuan (*n* = 216)	Guizhou (*n* = 127)	Chongqing (*n* = 65)
Sex					
Male	244 (45.60)	62 (48.82)	92 (42.59)	61 (48.03)	29 (44.62)
Female	291 (54.40)	65 (51.18)	124 (57.41)	66 (51.97)	36 (55.38)
Age					
Under 18 years	26 (4.86)	9 (7.09)	10 (4.63)	3 (2.36)	4 (6.16)
18 years	323 (60.37)	75 (59.06)	140 (64.81)	61 (48.03)	47 (72.31)
19 years	153 (28.60)	36 (28.35)	55 (25.46)	49 (38.58)	13 (20.00)
Over 19 years	33 (6.17)	7 (5.51)	11 (5.09)	14 (11.02)	1 (1.54)

### 3.2 Respondent’s personality traits, learning orientation, and decision-making style

The average scores of respondents on the Big Five Personality Test, Achievement Goal Questionnaire, and Rational-Experiential Inventory are illustrated in Table 2. The results were obtained through correlation analysis and binary logistic regression analysis, there is no statistically significant difference in the Big Five personality traits was found among respondents from different regions (*p* > 0.05). However, respondents from colleges and universities in Chongqing exhibited a significant difference in “mastering avoidance goals” on the Achievement Goal Questionnaire (*p* < 0.05), with scores notably higher than those in other regions. Conversely, no significant difference was found among respondents from various provinces and cities (*p* > 0.05).

**Table 2:** Analysis of respondents’ personality characteristics, et al.

Variable	Yunnan (*n* = 127)	Sichuan (*n* = 216)	Guizhou (*n* = 127)	Chongqing (*n* = 65)	Mean	*p*
Big five personality						
Adaptability	14.06 ± 2.83	13.24 ± 3.16	13.68 ± 3.07	14.05 ± 3.50	13.64 ± 3.12	0.075
Sociability	15.35 ± 2.89	15.02 ± 3.36	15.61 ± 2.86	15.62 ± 3.57	15.31 ± 3.17	0.305
Openness	14.54 ± 2.79	14.19 ± 2.92	13.79 ± 2.69	14.38 ± 3.22	14.20 ± 2.88	0.205
Altruism	17.75 ± 2.83	18.19 ± 3.29	18.30 ± 3.15	18.49 ± 3.12	18.15 ± 3.14	0.365
Moral sense	16.50 ± 2.84	16.83 ± 3.11	17.02 ± 2.66	17.34 ± 2.88	16.86 ± 2.92	0.247
Total	15.64 ± 1.57	15.49 ± 1.51	15.68 ± 1.22	15.98 ± 2.25	15.63 ± 1.57	
Achievement-goal						
Master the avoidance target	4.63 ± 1.12	4.54 ± 1.22	4.82 ± 1.11	4.95 ± 1.14	4.68 ± 1.17	0.032*
Grasp the approach target	5.67 ± 0.98	5.79 ± 0.97	5.76 ± 0.96	5.83 ± 0.93	5.76 ± 0.96	0.669
Achievement avoidance target	4.46 ± 1.20	4.45 ± 1.41	4.78 ± 1.12	4.75 ± 1.40	4.57 ± 1.30	0.062
Achievement approaches to target	4.98 ± 1.10	5.15 ± 1.17	5.07 ± 1.12	5.43 ± 1.19	5.12 ± 1.15	0.076
Total	4.94 ± 0.72	4.98 ± 0.79	5.11 ± 0.72	5.24 ± 0.86	5.03 ± 0.77	
Rational experience						
Faith in intuition	3.43 ± 0.65	3.41 ± 0.66	3.38 ± 0.61	3.53 ± 0.72	3.42 ± 0.65	0.509
Faith thinking	2.64 ± 0.58	2.78 ± 0.58	2.66 ± 0.48	2.70 ± 0.61	2.71 ± 0.56	0.111
Total	3.03 ± 0.40	3.09 ± 0.40	3.02 ± 0.36	3.23 ± 0.61	3.11 ± 0.49	

**Correlation is significant at the 0.01 level (2-tailed); *Correlation is significant at the 0.05 level (2-tailed).

### 3.3 Respondents’ motivations for studying clinical pharmacy

As shown in Table 3, among the respondents, scores between different regions on three items—“I am interested in clinical pharmacy,” “I am inspired by the guidance of clinical pharmacists,” and “I can choose other majors, but I do not know what they can do”—considerably differed (*p* < 0.05). On average, respondents tended to show a greater preference for choices such as “I want to have my own business,” “I want to work in a medical institution,” and “I want to work with clinicians.” Conversely, options like “I can choose other majors, but I do not know what they can do,” “My friends study in the medical field,” and “I want to carry on the family tradition” were relatively less popular compared to the average.

**Table 3:** Motivations of respondents from different regions to study clinical pharmacy.

Variable	Yunnan (*n* = 127)	Sichuan (*n* = 216)	Guizhou (*n* = 127)	Chongqing (*n* = 65)	Mean	*p*
I want a job where I can care about and help others	3.90 ± 0.85	4.02 ± 0.82	3.91 ± 0.79	3.88 ± 0.80	3.95 ± 0.82	0.390
I am interested in clinical pharmacy	3.20 ± 0.86	3.46 ± 0.89	3.35 ± 0.80	3.18 ± 1.01	3.34 ± 0.89	0.020*
I am interested in applying pharmacy to daily life	3.57 ± 0.95	3.79 ± 0.92	3.76 ± 0.79	3.60 ± 0.95	3.71 ± 0.91	0.097
I want to work in a medical institution	4.14 ± 0.75	4.19 ± 0.87	4.04 ± 0.82	4.17 ± 0.74	4.14 ± 0.82	0.448
I am interested in practicing clinical pharmacy technology	3.89 ± 0.83	3.94 ± 0.89	3.87 ± 0.75	3.77 ± 0.95	3.90 ± 0.85	0.543
I want to work with clinicians	4.17 ± 0.82	4.08 ± 0.87	3.94 ± 0.70	4.02 ± 0.94	4.06 ± 0.83	0.142
I am inspired by the guidance of clinical pharmacists	3.24 ± 1.01	3.53 ± 1.08	3.46 ± 0.90	3.26 ± 1.02	3.41 ± 1.02	0.037*
I want to have my own business	4.05 ± 0.88	4.19 ± 0.80	4.08 ± 0.82	4.28 ± 0.67	4.14 ± 0.82	0.171
I want to work in clinical pharmacy research	3.76 ± 0.91	3.77 ± 0.97	3.79 ± 0.79	3.65 ± 1.01	3.76 ± 0.92	0.767
I am attracted by the publicity of this university	3.02 ± 1.02	3.20 ± 0.98	3.12 ± 0.91	3.23 ± 1.01	3.14 ± 0.98	0.311
My parents wanted me to go into clinical pharmacy	2.92 ± 1.13	3.07 ± 1.00	3.02 ± 1.07	3.00 ± 1.06	3.02 ± 1.05	0.659
I could choose other majors, but I do not know what they would do	3.05 ± 1.08	2.85 ± 1.09	3.20 ± 1.05	3.08 ± 1.16	3.01 ± 1.09	0.033*
My friend studies in the medical field	2.87 ± 1.20	2.77 ± 1.22	2.85 ± 1.16	2.91 ± 1.16	2.83 ± 1.19	0.820
I want to carry on the family tradition	2.23 ± 1.09	2.26 ± 1.17	2.03 ± 1.02	2.43 ± 1.22	2.22 ± 1.13	0.106
Total	3.43 ± 0.53	3.51 ± 0.57	3.46 ± 0.49	3.46 ± 0.60	3.47 ± 0.55	

**Correlation is significant at the 0.01 level (2-tailed); *Correlation is significant at the 0.05 level (2-tailed).

### 3.4 Respondents’ interest in clinical pharmacy practice

Table 4 reveals significant regional differences among respondents in clinical pharmacy practices (*p* < 0.05), particularly in terms of “drug management,” “community services and science education,” and “sales of (over-the-counter) drugs.” Statistically, students in the Guizhou Province exhibited higher engagement in drug management, community services, popular science education, and sales of (over-the-counter) drugs than did students in other regions. From an average standpoint, “Consultation and medication consultation,” “Clinical pharmacy research,” and “Drug therapy monitoring” are above the average, while “Sales (over-the-counter) drugs,” “Drug dispensing and distribution,” and “Community services and popular science education” were below the average.

**Table 4:** Respondents’ interest in clinical pharmacy practice in different regions.

Variable	Yunnan (*n* = 127)	Sichuan (*n* = 216)	Guizhou (*n* = 127)	Chongqing (*n* = 65)	Mean	*p*
Drug dispensing and distribution	3.34 ± 1.03	3.35 ± 1.13	3.54 ± 0.90	3.20 ± 1.13	3.37 ± 1.06	0.174
Review medical orders	3.52 ± 0.94	3.58 ± 1.06	3.70 ± 0.84	3.54 ± 1.05	3.59 ± 0.98	0.480
Medication education and guidance for doctors and patients	3.77 ± 0.84	3.83 ± 1.01	3.89 ± 0.77	3.72 ± 0.98	3.82 ± 0.91	0.600
Consultation and medication consultation	3.87 ± 0.77	3.97 ± 0.94	4.06 ± 0.70	3.82 ± 0.92	3.95 ± 0.85	0.155
Drug management	3.31 ± 1.00	3.46 ± 1.04	3.72 ± 0.77	3.18 ± 1.00	3.45 ± 0.98	0.001**
Preparing medications	3.77 ± 0.80	3.79 ± 1.02	3.91 ± 0.80	3.63 ± 0.93	3.79 ± 0.91	0.226
Community service and popular science education	3.47 ± 0.98	3.36 ± 1.06	3.63 ± 0.84	3.20 ± 1.05	3.43 ± 1.00	0.020*
Selling (over-the-counter) medicines	3.15 ± 1.09	3.01 ± 1.18	3.35 ± 0.96	2.94 ± 1.12	3.12 ± 1.11	0.027*
Research in clinical pharmacy	4.02 ± 0.75	3.98 ± 0.96	3.93 ± 0.81	3.83 ± 0.91	3.96 ± 0.87	0.507
Drug therapy monitoring	3.87 ± 0.84	3.99 ± 0.89	4.04 ± 0.72	3.80 ± 0.85	3.95 ± 0.84	0.149
Total	3.61 ± 0.63	3.63 ± 0.74	3.78 ± 0.58	3.49 ± 0.74	3.64 ± 0.68	

**Correlation is significant at the 0.01 level (2-tailed); *Correlation is significant at the 0.05 level (2-tailed).

### 3.5 Respondents’ expectations for clinical pharmacy careers

Table 5 illustrates significant regional differences among respondents in their expectations for a clinical pharmacy career (*p* < 0.05), particularly in the aspect of “I hope to be able to take on work independently.” Notably, respondents from the Sichuan Province expressed greater expectations in this regard than did students from other provinces and cities. On average, expectations such as, “I hope to have a stable job,” “I hope to be able to find a job smoothly,” and “I hope to be respected in society” exceed the average. Conversely, expectations regarding “I hope to have my own pharmacy” and “I hope to work in a pharmaceutical company” were below the average.

**Table 5:** Expectations of clinical pharmacy careers in different regions.

Variable	Yunnan (*n* = 127)	Sichuan (*n* = 216)	Guizhou (*n* = 127)	Chongqing (*n* = 65)	Mean	*p*
I hope to become a professional clinical pharmacist	4.04 ± 0.80	4.06 ± 0.95	4.13 ± 0.77	3.92 ± 0.97	4.05 ± 0.88	0.473
I would like to be able to work as part of a clinical team	4.25 ± 0.72	4.24 ± 0.87	4.25 ± 0.70	4.22 ± 0.87	4.24 ± 0.80	0.989
I hope to be able to find a job smoothly	4.44 ± 0.64	4.50 ± 0.77	4.52 ± 0.58	4.45 ± 0.59	4.48 ± 0.67	0.768
I hope to have a stable job	4.43 ± 0.67	4.50 ± 0.73	4.57 ± 0.57	4.48 ± 0.59	4.50 ± 0.67	0.455
I hope to engage in health-related work	4.20 ± 0.75	4.26 ± 0.83	4.26 ± 0.67	4.23 ± 0.82	4.24 ± 0.77	0.892
I hope to have opportunities and channels to continue learning	4.39 ± 0.64	4.39 ± 0.75	4.32 ± 0.60	4.31 ± 0.73	4.36 ± 0.69	0.732
I hope to work in a medical facility	4.25 ± 0.73	4.33 ± 0.81	4.37 ± 0.60	4.32 ± 0.77	4.32 ± 0.74	0.633
I hope to be respected in society	4.40 ± 0.68	4.44 ± 0.72	4.35 ± 0.64	4.43 ± 0.59	4.41 ± 0.68	0.715
I hope to be well rewarded	4.37 ± 0.71	4.33 ± 0.75	4.31 ± 0.70	4.40 ± 0.63	4.34 ± 0.72	0.832
I want to have my own pharmacy	3.54 ± 0.84	3.48 ± 1.02	3.73 ± 0.86	3.54 ± 0.94	3.56 ± 0.94	0.107
I hope to be able to work independently	4.20 ± 0.71	4.23 ± 0.79	4.17 ± 0.65	3.82 ± 0.88	4.16 ± 0.76	0.001
I hope to work in a pharmaceutical company	3.94 ± 0.76	3.75 ± 0.99	3.93 ± 0.89	3.83 ± 0.93	3.85 ± 0.91	0.194
I hope to be responsible for patients	4.30 ± 0.65	4.39 ± 0.74	4.43 ± 0.62	4.22 ± 0.76	4.36 ± 0.70	0.155
Total	4.21 ± 0.47	4.22 ± 0.61	4.26 ± 0.47	4.17 ± 0.53	4.22 ± 0.53	

**Correlation is significant at the 0.01 level (2-tailed); *Correlation is significant at the 0.05 level (2-tailed).

## 4 Discussion

The findings revealed no significant regional differences in the propensity to change majors, transfer to this major, pursue related careers post-graduation, or in the personality traits across different regions. However, considerable disparities were observed among respondents from various regions regarding their motivation for the pharmacy profession, interest in clinical pharmacy practice, career expectations in clinical pharmacy, and achievement goals. Notably, the Big Five Personality Test showed no significant difference in the personality traits of the respondents in the four provinces and cities in southwest China. However, respondents from Chongqing exhibited considerably higher scores than those from other regions in “mastering avoidance goals, “indicating a tendency to avoid undesirable outcomes, often accompanied by feelings of nervousness, anxiety, and fear (19).

Based on these findings, university pharmacy schools can utilize the concept of “Mastery of Avoidance Goals” to attend to the personal circumstances of college freshmen. Regular questionnaire surveys on students’ psychology and career planning can facilitate a proactive understanding of their future and psychological conditions (20). This approach enables college freshmen to develop skills and abilities to navigate away from negative outcomes and judgment, thus emphasizing the humane aspect of university pharmacy schools. According to the research of Noble Po-Kan Lo et al., teachers’ sense of happiness can also be an important factor affecting students’ motivation and the educational environment (21). Applying positive psychology interventions such as mindfulness and gratitude to the training of pharmacy teachers may be a promising direction for optimizing educational practices in the future.

Research conducted in China suggests that strengthening education on new knowledge in clinical pharmacy, increasing the professionalism of clinical pharmacy courses, and engaging new students to envision the potential in the field can lead to higher learning achievements (22). By doing so, students can develop the ability to shift from initial goals to mastery and approach goals effectively.

The Rational-Experiential Inventory showed no significant difference among respondents from four provinces and cities in southwest China. Regarding the motivation for studying clinical pharmacy, among the respondents, “I am interested in clinical pharmacy,” “I am inspired by the guidance of clinical pharmacists,” and “I can choose other majors, but I do not know what they can do.” On the three items, significant differences were present in scores between different regions. Specifically, respondents from colleges and universities in the Sichuan Province were significantly higher than those from other provinces and cities in terms of “I am interested in clinical pharmacy” and “I am inspired by the guidance of clinical pharmacists,” probably owing to the original characteristics of the Sichuan Province. West China University of Medical Sciences was the pioneer in enrolling clinical pharmacy students in 1989, initiating modern clinical pharmacy education in China and shaping student training in the field (19). Despite its merger with the Sichuan University, the institution, alongside West China Hospital, continues to wield considerable influence in medicine across the Sichuan Province, the southwest region, and even nationally, attracting students to its programs (23).

The respondents’ preferences for career paths, such as owning their practice, working in medical institutions, and collaborating with clinicians, exceed the average, indicating disparities in training approaches between China and foreign countries. While China’s clinical pharmacy professional model emphasizes roles in hospitals and medical institutions, respondents also express interest in pharmaceutical companies. This trend differs among the Western countries such as the United States and the United Kingdom, where clinical pharmacy students often pursue diverse paths, including community pharmacy roles (13, 24).

However, respondents exhibited limited awareness of clinical pharmacy, possibly due to a lack of familial and social exposure to clinical pharmacy profession. This is evidenced by their relatively low consideration of factors like familial traditions and friends studying in the medical field. To address this, universities and clinical pharmacists should increase promotional efforts through various channels, such as newspapers, the internet, and community activities to improve the public awareness, stimulate student motivation, and promote clinical pharmacy development (25, 26).

Respondents from different regions showed significant differences in clinical pharmacy practice in terms of “drug management,” “community services and science education,” and “sales of (over-the-counter) drugs.” This contrasts with the findings of Nair et al. (18), who found that students at the University of Waterloo in Canada prioritized patient interaction, health promotion, drug counseling, and continuing professional development. Conversely, students at the University of Otago in New Zealand showed higher interest in patient physical examinations, drug synthesis, and research. In this study, students in Guizhou Province have a higher level of participation, which may be due to policy reasons. In recent years, China’s strategic development under the government has led to extraordinary development in the field of smart healthcare for Guizhou Province. This industrial prospect paints a highly attractive future career picture for pharmacy students in clinical pharmacy practice (27). There’s also a possibility that it’s because higher education institutions place importance on the cultivation of clinical pharmacy practice for students.

Foreign research suggests that students prioritize patient-centered care (28), where this study indicates that respondents in southwest China place greater emphasis on the technical aspects of clinical pharmacy. Notably, “Consultation and medication consultation,” “Clinical pharmacy research,” and “Drug therapy monitoring” scored higher than average, while “Sales of (non-prescription) drugs,” “Drug dispensing and distribution,” and “Community services and science Education” scored lower.

Respondents displayed a preference for hospital-based clinical pharmacy skills and research, with comparatively less interest in community or pharmacy aspects such as drug distribution, sales, and science popularization. Incorporating foreign experience into the school’s clinical pharmacy training syllabus to increase the knowledge points of humanistic education, and focusing on people-oriented clinical pharmacy skills training in practical training will help improve students’ abilities in related fields. Additionally, providing opportunities for students to personally experience clinical pharmacy practices in training bases can deepen their understanding of the clinical pharmacy profession (29).

Significant differences were observed among respondents from different regions regarding their expectations of being able to work independently in their future clinical pharmacy careers. On average, aspirations for job stability, smooth employment opportunities, and societal respect scored higher than the average. These findings align with those of Nair et al. (18), who noted similar priorities among students at the University of Waterloo in Canada and the University of Otago in New Zealand, high ling the universal importance of job stability and professional recognition.

The lower average values for aspirations to own a pharmacy or work in a pharmaceutical company indicate a lesser interest in these career paths among respondents. This suggests that students in this study were more focused in finding stable and respected positions rather than entrepreneurial or corporate jobs. Given that students are in a phase of career exploration during their university years, their awareness of career planning considerably influences their future career choices and development (30).

To address this aspect, the School of Pharmacy can integrate employment and career guidance courses into the curriculum, either as required or elective courses. This can help students to understand their career goals and positioning, ultimately facilitating smoother transitions into the workforce (24). By providing structured support for career exploration and development, the school can empower students to make informed decisions about their future paths in clinical pharmacy.

The admission standards and selection mechanism for undergraduate clinical pharmacy students in southwest China are undergoing continuous adjustments and optimizations, including measures such as the integration of the college entrance examination (31). These changes reflect the ongoing deepening of education reform in China and pose challenges to traditional higher education selection models. Colleges and universities are striving to adopt more scientific, fair, and effective methods to select and train future clinical pharmacy talents.

While China’s enrollment reform path may be fraught with challenges, high-quality pharmaceutical talents with profound professional knowledge, innovative abilities, and practical skills should be cultivated to meet the needs of social development and medical and health care (31, 32). By prioritizing the cultivation of such talents, institutions can contribute significantly to advancing medical and healthcare services and addressing the complex needs of society (33).

Furthermore, the link between educational practice and local industry should be strengthened, adjusting professional curricula according to align with the needs of the regional job market. This approach aims to enhance students’ professional competitiveness, meet societal and economic demands, and advance the continuous improvement of clinical pharmacy education and talent training (34, 35).

However, the research has certain limitations, such as the study’s limited scope and potential information bias due to the online questionnaire collection method. Moreover, the study’s focus on first-year undergraduate students, this limits its universality among students of other grades. This study also focuses only on students in the southwest region of China, which may have geographical limitations.

Future research can combine wider geographical areas, large-scale samples, diverse samples, and long-term data tracking to obtain more general and in-depth conclusions. Building upon the identified regional differences, future studies could explore strategies to optimize educational approaches across different regions and promote educational equity by integrating resources from various locales.

## 5 Conclusion

We examined the personality traits of freshmen majoring in clinical pharmacy in southwest China and found distinct regional and emerging characteristics amidst ongoing education reforms. Our study reports significant differences in respondents’ motivation for studying clinical pharmacy, their interests in clinical pharmacy practice, and their career expectations, shedding light on the influence of regional disparities on respondents’ personality. These findings offer valuable insights for formulating more precise teaching strategies and training plans.

Pharmacy schools in universities are encouraged to focus on the characteristics and career plans of freshmen (35), and help to ensure that they have received professional training in designing courses or other aspects, laying a foundation for the training of professional clinical pharmacy talents in the future.

## Data Availability

The original contributions presented in the study are included in the article/Supplementary material, further inquiries can be directed to the corresponding authors.

## Footnotes

^1^ https://www.wjx.cn

^2^ https://www.ibm.com/spss
